# Robust Data-Driven Leak Localization in Water Distribution Networks Using Pressure Measurements and Topological Information

**DOI:** 10.3390/s21227551

**Published:** 2021-11-13

**Authors:** Débora Alves, Joaquim Blesa, Eric Duviella, Lala Rajaoarisoa

**Affiliations:** 1 Supervision, Safety and Automatic Control Research Center (CS2AC), Universitat Politècnica de Catalunya, Gaia Building, Rambla Sant Nebridi, 22, 08222 Terrassa, Spain; 2 IMT Nord Europe, Université de Lille, CERI Digital Systems, F-59000 Lille, France; eric.duviella@imt-lille-douai.fr (E.D.); lala.rajaoarisoa@imt-lille-douai.fr (L.R.); 3 Institut de Robòtica i Informàtica Industrial (CSIC-UPC), Carrer Llorens Artigas, 4-6, 08028 Barcelona, Spain; 4 Serra Húnter Fellow Automatic Control Department (ESAII), Universitat Politècnica de Catalunya (UPC), Pau Gargallo, 5, 08028 Barcelona, Spain

**Keywords:** water distribution networks, leak localization, data-driven

## Abstract

This article presents a new data-driven method for locating leaks in water distribution networks (WDNs). It is triggered after a leak has been detected in the WDN. The proposed approach is based on the use of inlet pressure and flow measurements, other pressure measurements available at some selected inner nodes of the WDN, and the topological information of the network. A reduced-order model structure is used to calculate non-leak pressure estimations at sensed inner nodes. Residuals are generated using the comparison between these estimations and leak pressure measurements. In a leak scenario, it is possible to determine the relative incidence of a leak in a node by using the network topology and what it means to correlate the probable leaking nodes with the available residual information. Topological information and residual information can be integrated into a likelihood index used to determine the most probable leak node in the WDN at a given instant *k* or, through applying the Bayes’ rule, in a time horizon. The likelihood index is based on a new incidence factor that considers the most probable path of water from reservoirs to pressure sensors and potential leak nodes. In addition, a pressure sensor validation method based on pressure residuals that allows the detection of sensor faults is proposed.

## 1. Introduction

Water distribution networks are complex systems that are difficult to manage and monitor with extreme importance nowadays. The detection and location of leaks have become crucial for water distribution because when there are bursts or leaks, this can generate not only economic losses but also an environmental issue and represents a potential risk to public health with contaminated water [1]. Another concern is the scarcity of water that can occur in 2025, which may affect half the world’s population that will not have access to safe and accessible water for their basic needs [2]. However, with all these risks, currently, this infrastructure does not perform satisfactorily in practice. According to [3], a global volume of water loss called Non-Revenue Water (NRW) has been calculated at 346 million cubic meters per day or 126 billion cubic meters per year.

The infrastructure in a medium-sized city can have pipes that span hundreds of kilometers connected to hundreds of nodes (pipe junctions or customers that connect to the network). Therefore, several factors can generate water loss during transport between the treatment plants and the reservoir for consumers, usually attributed to several causes, including leaks, measurement errors, and theft. Water loss can be divided into two terms, “real losses” and “apparent losses”. Apparent losses are constituted by badly read measurements, data handling errors, and illegal water tapping. In contrast, the real losses comprise leakage from all system parts and overflow at storage tanks. Real losses are divided into “background leakage” made up of small undetectable and into detectable leaks relevant for detection as they represent significant losses for the water distribution company.

Effective leak management is vital for all of the factors mentioned above to save financial resources and water. The methods of leak localization can be classified into two categories: Hardware-based system and Software-based.

The Hardware-based utilizes hardware sensors to detect a leak directly and help the localization of the leak. As there are various types of sensors and instruments available, they can be further subclassified as: acoustic [4,5] and non-acoustic detection methods [6].

Software-based methods generally rely on an algorithm or model for detecting leaks. Unlike hardware-based methods, these methods do not seek to locate the leak point accurately but minimize possible leakage areas. Since these methods are based on information, such as the pressure of the pipe network, flow data, and so forth, they work well on any type of pipe. These methods can be divided into physical modeling methods and data-driven methods. The physical modeling methods or model-based methods identify the leak using a numerical model and compare the results with the field data, for example, Ref. [7] which uses pressure sensitivity analysis, Ref. [8] uses leak signature space, Ref. [9] analyzes the sensitivity matrix and residuals, and [10] uses pressure and flow measurements to perform leakage detection through model-invalidation. On the other hand, data-driven methods analyze the monitoring data, combining tools such as artificial intelligence (e.g., classifiers [11,12,13,14] or artificial neural networks [15,16]). Thus, it is possible to identify potential areas of the leak based on certain rules or principles without resorting to the simulation of the physical model results [17]. However, these methods need, in general, an important number of non-leak and leak data scenarios in the training process to obtain reasonable results. As an exhaustive amount of leak scenarios are not available in general, a hydraulic simulator can be used to generate leak data. This work deals with the problem of leak localization and it is assumed that it is available a leak detection method that determines if a leak is present or not in the WDN. In particular, a non-numerical localization method, focused on a data-driven approach, is proposed.

Like other recent works [18,19], it requires only topological information of the network and historical data without leakage of the available measurements. In this work, the topological information provides the most probable paths for extra flows produced by leaks. A new incidence factor from every combination of nodes and sensors is computed with this information. Every incidence factor determines how a leak in a particular node affects a specific pressure sensor. On the other hand, historical data are used to calculate non-leak pressure estimations at sensed inner nodes. Residuals are generated using the comparison between these estimations and leak pressure measurements. Incidence factors are integrated with residuals in likelihood indexes to give the most probable leak node in a leak scenario. In addition, pressure residuals are used to detect sensor faults by means of a novel sensor validation algorithm.

The remainder of this paper is organized as follows: Section 2 presents the theory of graphs applied to WDN and explains the structure of the reduced-order model used in this work. The developed leak localization has been elaborated in Section 3. In Section 4 a sensor validation method that allows the detection of pressure sensor faults is presented. Section 5 introduces the case studies of Hanoi and Modena’s WDNs. Section 6 presents the conclusions and future scope of the research work.

## 2. Water Distribution Networks

### 2.1. Preliminaries

A water distribution network is composed of *m* pipes, *n* internal consumer nodes and can be described by a directed graph G={V,E},[20], with $V=\{v_{1},\ldots ,v_{n}\}$ is the set of vertices that represent connections between the components of the network, additionally the last $\left\{v_{n-n_{I}+1},\ldots ,v_{n}\right\}$, represent the vertices of the system’s input, $n_{I}$ being the number of the inlets, with $n_{I}\geq 1$. The elements of the set $E=\{e_{1},\ldots ,e_{m}\}$ are the edges, which represent the *m* pipes in the network.

The graph G can be represented by the incidence matrix $H=[h_{ij}]$, in which the elements $h_{ij}$ are defined as:

$$h_{ij}=\left\{\begin{array}{ll} -1 & \mathrm{if}\;\mathrm{the}\;j\mathrm{th}\;\mathrm{edge}\;\mathrm{is}\;\mathrm{entering}\;i\mathrm{th}\;\mathrm{vertex}.\\ 0 & \mathrm{if}\;\mathrm{the}\;j\mathrm{th}\;\mathrm{edge}\;\mathrm{is}\;\mathrm{not}\;\mathrm{connected}\;\mathrm{to}\\ & \mathrm{the}\;i\mathrm{th}\;\mathrm{vertex}.\\ 1 & \mathrm{if}\;\mathrm{the}\;j\mathrm{th}\;\mathrm{edge}\;\mathrm{is}\;\mathrm{leaving}\;i\mathrm{th}\;\mathrm{vertex}. \end{array}\right.$$

The direction of the edge represents a reference direction for the flow in the corresponding pipe. The incidence matrix is composed of $H\in {\left\{-1,0,1\right\}}^{n\times m}$ with each row corresponding to a node and column corresponding to a pipe.

The WDN must fulfill mass conservation law, which expresses the conservation of mass in each vertex, described by:

(1) H·q=d,

where $d\in R^{n}$ is the vector of nodal demands, with $d_{i}$ > 0 when the flow is into the node *i*, and $q\in R^{m}$ is the vector of flows in the edges. By virtue of the mass conservation, it is possible to have only n−1 independent nodal demand, $\sum_{i=1}^{n}d_{i}=0$, therefore the supply flow must equal the end-user demands as there is no storage in the network.

Let p be the vector of absolute pressures at the nodes and Δp be the vector of differential pressures across the pipes, both in meters of water column [mwc], then the energy law for water networks gives:

(2) $$\begin{array}{c} \Delta p=H^{T}p=f\left(q\right)-H^{T}h, \end{array}$$

where $p\in R^{n}$, and $f:R^{m}\to R^{m}$, $f\left(q\right)=(f_{1}\left(q_{1}\right),\ldots ,f_{m}\left(q_{m}\right))$. The function $f_{j}\left(\cdot \right)$ describes the flow dependent pressure drop due to the hydraulic resistance in the *j*th edge. The relationship between pipe flow and energy loss caused by friction in individual pipes can be computed using the Hazen–Williams formula [21] for expression $f_{j}\left(\cdot \right)$:

(3) $$f_{j}\left(q_{j}\right)=\frac{10.7\cdot L_{j}}{\rho_{j}^{1.852}\cdot D_{j}^{4.87}}\cdot q_{j}^{1.852},$$

where $L_{j}$ is the length of the pipe and $D_{j}$ is the diameter of the pipe, both in meters [m], $q_{j}$ is the pipe flow in m^3^/s and $\rho_{j}$ is the pipe roughness coefficient.

The term $H^{T}h$ is the pressure drop across the pipes due to the difference in geodesic level (i.e., elevation) in meters [m] between the ends of the pipes with $h\in R^{n}$ the vector of geodesic levels at each vertex.

### 2.2. Structure of the Reduced Order Model

The reduced-order network model is used in this paper to calculate the nominal pressure at the measured internal nodes. The model uses the pressure dependence of the network’s internal nodes with the pressure and flow measurements of the inlets. The details of the model derivation can be found in [22,23].

A network can be divided into nodes connected with reservoirs (the inlets nodes) and internal nodes that compose the system. To facilitate the explanation in this work, the information regarding inlet nodes will be represented by (r) superscript and those of the internal nodes, which will be expressed by the $\left(i_{n}\right)$ superscript. In particular, vector $p^{\left(in\right)}$ will contain pressure node values $p_{1},\ldots ,p_{n-n_{I}}$ and $p^{\left(r\right)}$ inlet pressure values $p_{n+1-n_{I}},\ldots ,p_{n}$.

The network needs to fulfill some conditions for using the reduced model proposed:

Condition 1: corresponds to the demands of the internal nodes of the system, where Equation (1) can be defined as:

(4) d(k)=−v(k)σ(k),

where σ(k) denotes the total inlet flow into the network at time instant *k*, the vector v(k) defines the distribution of the total demand in the internal nodes at every time *k*, with the property $\sum_{i}^{n}v_{i}\left(k\right)=1$. Notice that if all consumers are residential, all nodes demand have the same consumption profile, in consequence, the  v(k) will be constant v(k)=v.

Condition 2: is a particularly case when the vector $p^{\left(r\right)}$ of control inputs fulfill the following case,

(5) $$p^{\left(r\right)}\left(k\right)+h^{\left(r\right)}=\kappa \left(k\right)𝟙,$$

for some κ∈R, which is the total head at the inlets in [mwc] and where 𝟙 denote the vector consisting of ones. In [23], there is a discussion on this definition’s feasibility where the controllers should satisfy this premise at least in networks with the low total consumption.

If these two conditions are fulfilled, the pressure at the *i*th internal node can be expressed by:

(6) $$p_{i}^{\left(i_{n}\right)}\left(k\right)=\alpha_{i}\sigma^{2}\left(k\right)+\sum_{j=1}^{n_{I}}\beta_{ij}\left(k\right)p_{j}^{\left(r\right)}\left(k\right),$$

where $\alpha_{i}$ is parameter dependent on the network topology and the distribution of demands in the network, and $\beta_{ij}$ is dependent on the network topology with $j=1,\ldots ,n_{I}$. The total inlet flow σ is typically well-known since inlet flows are measured. $\alpha_{i}$ is a parameter dependent on the network topology and the distribution of demands in the network, and $\beta_{ij}$ is dependent on the network topology with $j=1,\ldots ,n_{I}$. The total inlet flow σ is typically well-known since inlet flows are measured.

Some methods of identifying parameters can be used to identify parameters $\alpha_{i}$ and $\beta_{ij}$ since model (6) of $p_{i}^{\left(i_{n}\right)}$ is linear [24], using the measures of σ, $p^{\left(r\right)}$ and $p_{i}^{\left(i_{n}\right)}$ with nodes that contain pressure sensors that will be denoted as $p_{s_{i}}$$\forall i=1,\ldots ,n_{s}$ in the following, where $n_{s}$ is the number of sensors installed in the inner nodes.

Once inner pressure model (6) has been calibrated, the accuracy of the model can be assessed by applying the computation of the model error or pressure residual defined by:

(7) $$r_{s_{i}}={\hat{p}}_{s_{i}}\left(c\right)-p_{s_{i}}\left(c\right),\;\forall i=1,\ldots ,n_{s},$$

where *c* denotes the boundary conditions (heads and inflows in inlets) necessary to compute pressure estimation by means of (6). For example, minimum and maximum residual bounds $\underline{\sigma_{i}}$ and ${\bar{\sigma }}_{i}$, considering the available data, can be computed for every sensor $i=1,\ldots ,n_{s}$ to obtain an idea of the accuracy of model (6). Sensor noises and error models can produce residual errors. If big values of residual bounds are obtained, improvements in model (6) should be considered. For example, the assumption that all the nodes have the same consumption profile can lead to a big error in some networks. In this case, the error could be decreased if model (6) is calibrated only using data from the same hour but on different days. It would be assumed that different users can have different profiles at a given hour, but a particular user will have the same profile at a particular hour for all the different days. This possible improvement will imply the calibration of 24 different models (6) (one for each hour) and will require more historical data to obtain good accuracy. Another method to obtain an estimation of the pressure in inner nodes ${\hat{p}}_{s_{i}}\left(c\right)$ is to use historical data directly as a lookup table, as was proposed in [18]. That is, given particular operating conditions, *c* provides the inner pressures from historical data that had the closest operating conditions $\hat{c}$ to *c*. Residuals (7) considering leak pressure measurements will be used in the leak localization, as will be explained in detail in the next section.

## 3. Leak Localization

The location of the leak on the WDN is typically divided into two steps: leak detection and leak localization [25]. The focus in this work is the leak localization assuming that the detection has already been effectuated. In addition, it is assumed that the leaks can only happen in the nodes of the network (as considered in [7,26], or [8]), making the number of nodes equal to the number of potential leaks. The nodes correspond to water users, pipe junctions, and other structures such as hydrants. However, if the number of nodes will not provide a representative discretization of the network, some artificial nodes could be considered.

In this Section, two leak localization methods will be proposed. The first one will only use available measurements, and its diagnosis will point to one of the inner pressure sensors installed in the WDN. Therefore, the detected leak should be in an area around this sensor (cluster). The second method will combine the information of the first method with the topological information: characteristics of the pipes and connections between the nodes of the WDN, in a likelihood index that will allow the leak localization at the node level.

### 3.1. Leak Localization at Cluster Level

As stated before, the proposed leak localization is applied after the leak detection. In addition to inlet pressure and flow sensors, it is assumed that $n_{s}$ pressure sensors are installed at different inner nodes. Consider a leak $l_{j}$ acting on the node *j* of the network, and the used measurements are assumed to be captured under a leaky situation. Additionally, admitting leak-free historical data of all the sensors are available. The residual pressure in internal nodes that contain a sensor, defined in (7), can be computed as:

(8) $$r_{s_{i}}={\hat{p}}_{s_{i}}\left(c\right)-p_{s_{i}}\left(c^{l_{j}}\right),\;\forall i=1,\ldots ,n_{s},$$

where ${\hat{p}}_{s_{i}}\left(c\right)$ is the pressure estimation considering boundary conditions *c* in a leak-free scenario. On the other hand, $p_{s_{i}}\left(c^{l_{j}}\right)$ is the pressure value measured by the inner pressure sensor *i* under boundary conditions $c^{l_{j}}$ (the same heads and inflows in inlets as in *c* but with a leak in node *j*).

Following the ideas in [18], positive residuals can be obtained from the following transformation:

(9) $${\bar{r}}_{s_{i}}=r_{s_{i}}-\min\left(r_{s_{1}},\ldots ,r_{s_{ns}}\right)\;\forall i=1,\ldots ,n_{s}.$$

Then, as the leak localization can be achieved by determining the residual pressure component with maximum size (see [22,27]), leak localization can be formulated as:

(10) $$\hat{J}=\underset{i\in \{1,\ldots ,n_{s}\}}{arg max}\left\{{\bar{r}}_{s_{i}}\right\}.$$

Notice that the result of the leak localization method (10) is one of the $n_{s}$ pressure sensor locations.

Then, the leak localization results in $\hat{J}$ point not only to sensor location $s_{j}$ but also to the nodes that produce a higher incidence for this sensor than the other sensors (cluster *j*).

### 3.2. Leak Localization at Node Level

Considering the Hazen–Willians Equation (3) for every pipe (edge $e_{z}$) a resistance $R_{z}$ can be defined:

(11) $$R_{z}=\frac{10.7\cdot L_{z}}{\rho_{z}^{1.852}\cdot D_{z}^{4.87}}.$$

Among the multiple pipe paths that can connect every pair of nodes ij, a path $P_{ij}^{min}$ with a minimum total resistance $R_{ij}$ can be computed by means of:

(12) $$P_{ij}^{min}=\underset{P_{ij}^{\left(k\right)}\in P_{ij}}{arg min}\sum_{e_{z}\in P_{ij}^{\left(k\right)}}R_{z},$$

where $P_{ij}=\left\{P_{ij}^{\left(r\right)},\ldots ,P_{ij}^{\left(e\right)}\right\}$ denotes the set of paths connecting nodes *i* and *j*.

On the other hand, the minimum path from the $n_{I}$ inlets to a node *j*, $I_{j}^{min}$, can be obtained by applying the computation of the minimum paths from the $n_{I}$ inlets to node *j* by means of (12) and determine which is the one with the minimum resistance among the $n_{I}$ paths.

When a leak is produced in node *j*, $I_{j}^{min}$ is the most probable path for the extra flow produced by the leak. So the effect of a leak in node *j* to sensor $s_{i}$ depends on the intersection of the paths from inlets to node *j* and the node where the sensor is located $s_{i}$: $I_{j}^{min}$ and $I_{s_{i}}^{min}$. To quantify the degree of incidence of the leak to the sensor, an incidence factor $g_{j,s_{i}}$ is defined as:

(13) $$g_{j,s_{i}}=R_{j,s_{i}}^{c}{\bar{g}}_{j,s_{i}},$$

where $R_{j,s_{i}}^{c}$ is the resistance of the path defined by $I_{j}^{min}\cap I_{s_{i}}^{min}$, the superscript *c* refers to the common path between node and sensors, and ${\bar{g}}_{j,s_{i}}$ is a normalization factor that takes into account the inverse of the resistance from the node *j* to the different sensors:

$${\bar{g}}_{j,s_{i}}=\left\{\begin{array}{ll} \frac{\frac{1}{R_{j,s_{i}}}}{\sum_{l=1}^{n_{s}}\frac{1}{R_{j,s_{i}}}} & \mathrm{if}\;j\neq s_{i}\\ 1 & \mathrm{if}\;j\;=\;s_{i}. \end{array}\right.$$

The $n_{s}$ incidence factors associated to a leak in node *j*, $g_{j,s_{i}}$$i=1,\ldots ,n_{s}$ can be normalized:

(14) $$\lambda_{j,s_{i}}=\frac{g_{j,s_{i}}}{\sum_{l=1}^{n_{s}}g_{j,s_{l}}},$$

where coefficient $\lambda_{j,s_{i}}$ determines the relative incidence of a leak in node *j* to sensor $s_{i}$ regarding all the $n_{s}$ sensors and the need to fulfill:

(15) $$\sum_{i=1}^{n_{s}}\lambda_{j,s_{i}}=1\;\forall j=1,\ldots ,n-n_{I}.$$

For every node $j=1,\ldots ,n-n_{I}$, the most sensitive sensor to a leak in this node can be computed as:

(16) $$\hat{J}=\underset{i\in \{1,\ldots ,n_{s}\}}{arg max}\left\{\lambda_{j,s_{i}}\right\}.$$

The $n_{s}$ clusters used in the leak localization defined in (10) can be computed using the set of nodes that provide the same value of $\hat{J}$. The following equation is the definition of the cluster associated with the sensed node *l*:

(17) $$C_{l}=\left\{v_{j}\in V|\underset{i\in 1,\ldots ,n_{s}}{arg max}\left\{\lambda_{j,s_{i}}\right\}=l\right\},$$

where $l=1,\ldots ,n_{s}$. The topological information of $\lambda_{j,s_{i}}$ and the measurement information of residuals ${\bar{r}}_{s_{i}}$ can be integrated in a parameter $\theta_{j}$ defined as:

(18) $$\theta_{j}=\frac{1}{\bar{\theta }}\sum_{i=1}^{n_{s}}\lambda_{j,s_{i}}{\bar{r}}_{s_{i}}$$

where $\bar{\theta }$ is a normalization factor. Then, $\theta_{j}$ can be interpreted as a likelihood index, and the leak localization at cluster level defined in (10) can be formulated at node level as:

(19) $$\hat{J}=\underset{j\in \{1,\ldots ,n-n_{I}\}}{arg max}\left\{\theta_{j}\right\}.$$

In order to improve the performance of the leak localization method, the information of the residuals at different time instants *k* can be taken into account applying the Bayes’ rule as:

(20) $$P_{j}\left(k\right)=\frac{P_{j}\left(k-1\right)\theta_{j}\left(k\right)}{\sum_{l=1}^{n-n_{I}}P_{l}\left(k-1\right)\theta_{l}\left(k\right)},$$

where $P_{j}\left(k-1\right)$ is the prior probability whose initial value $P_{j}\left(k-1\right)$ has to be determined (for example $P_{j}\left(0\right)=1/\left(n-n_{I}\right)$). Then, the leak node localization can be estimated by using posterior leak probabilities by:

(21) $$\hat{J}\left(k\right)=\underset{j\in \{1,\ldots ,n-n_{I}\}}{arg max}\left\{P_{j}\left(k\right)\right\}.$$

## 4. Sensor Validation

When a leak is not detected by the leak detection method, anomalous values of pressure residuals $r_{s_{i}}\left(k\right)$$i=1,\ldots ,n_{s}$ defined in (7) can be used to detect sensor faults. In the same operating conditions, the historical data of inner pressure sensors (leak-free data or data for a particular leak scenario) can be used first to calibrate a pressure estimation model as described in Section 2.2. Secondly, to determine residual bounds $\underline{\sigma_{i}}$ and ${\bar{\sigma }}_{i}$ that allows the implementation of pressure sensor fault detection through checking:

(22) $$\left\{\begin{array}{c} r_{s_{i}}\left(k\right)\in \left[\underline{\sigma_{i}},{\bar{\sigma }}_{i}\right]\Rightarrow \;\mathrm{No}\;\mathrm{Fault}\;(\phi_{i}\left(k\right)=0)\\ r_{s_{i}}\left(k\right)\notin \left[\underline{\sigma_{i}},{\bar{\sigma }}_{i}\right]\Rightarrow \mathrm{Fault}\;\mathrm{in}\;\mathrm{sensor}\;s_{i}\;(\phi_{i}\left(k\right)=1). \end{array}\right.$$

The accuracy of this fault detection method depends on the length of residual bounds $\underline{\sigma_{i}}$ and ${\bar{\sigma }}_{i}$ and, therefore, on the accuracy of pressure estimation (6). In order to increase the accuracy of the fault detection method, spatial residuals [28] between pressure residuals (7) can be computed

(23) $$Sr_{s_{i},s_{j}}\left(k\right)=r_{s_{i}}\left(k\right)-r_{s_{j}}\left(k\right)\;\forall i=1,\ldots ,n_{s}-1\;\mathrm{and}\;j=i+1,\ldots ,n_{s}.$$

In the same way as the pressure residuals, spatial residual bounds ${\underline{\varepsilon }}_{i,j}$ and ${\bar{\varepsilon }}_{i,j}$ can be computed using leak-free data, and the fault detection can be implemented as follows:

(24) $$\left\{\begin{array}{c} Sr_{s_{i},s_{j}}\left(k\right)\in \left[{\underline{\varepsilon }}_{i,j},{\bar{\varepsilon }}_{i,j}\right]\Rightarrow \mathrm{No}\;\mathrm{Fault}\left(\Phi_{i,j}\left(k\right)=0\right)\\ Sr_{s_{i},s_{j}}\left(k\right)\notin \left[{\underline{\varepsilon }}_{i,j},{\bar{\varepsilon }}_{i,j}\right]\Rightarrow \mathrm{Fault}\left(\Phi_{i,j}\left(k\right)=1\right). \end{array}\right.$$

As model errors will affect in a similar way as close pressure sensors, it is expected that some spatial residual bounds will be smaller than pressure residual bounds. Therefore, fault detection defined by (24) will be more sensitive to pressure sensor faults than the one defined by (22). The accuracy of the sensor fault detection can be increased by means of average computing residuals in a time window leading to smaller residual bounds.

Once a residual has been violated, that is, at least one of the sensor faulty signals $\phi_{i}\left(k\right)$$i=1,\ldots ,n_{s}$ or spatial faulty signals $\Phi_{i,j}\left(k\right)\;$$i=1,\ldots ,n_{s}-1\;\mathrm{and}\;j=i+1,\ldots ,n_{s}\;$ is equal to one, the sensor fault isolation can be implemented in two stages as described in Algorithm 1:
**Algorithm 1** Sensor validation search for sensor fault*Stage 1:* In the case of the activation of one or more sensor faulty signals $\phi_{i}\left(k\right)$$i=1,\ldots ,n_{s}$, as these signals are uniquely related to sensors $s_{i}$$i=1,\ldots ,n_{s}$, the isolation is trivial and faulty sensors must be discarded for future leak localization, and the number of available healthy sensors $n_{s}$ should be updated.*Stage 2:* Only considers Spatial faulty signals $\Phi_{i,j}\left(k\right)$ of the $n_{s}$ non-faulty sensors from Stage1. As these fault signals are potentially affected by two possible sensor faults $s_{i}$ and $s_{j}$, the fault isolation can be implemented iteratively by the following steps:
1: **for**
$i\leftarrow 1,n_{s}-1$
**do**

2:  **for**
$j\leftarrow i+1,n_{s}$
**do**

3:   **for**
$\Phi_{i,j}\left(k\right)==1$
**then**

4:
(25)$$\hat{ı}=\underset{i\in \{1,\ldots ,n_{s}\}}{arg max}\left\{\sum_{j=i+1}^{n_{s}-1}\Phi_{i,j}\left(k\right)+\sum_{j=1}^{i-1}\Phi_{j,i}\left(k\right)\right\}.$$

5:    Discard sensor $s_{\hat{ı}}$, eliminate faulty signals related to this sensors, update $n_{s}$.

6:   **end if**

7:  **end for**

8: **end for**


In the case that two or more sensors obtain the same cost function in (25) and less than the maximum possible value $n_{s}-1$, the computation of (25) should be performed in a time window until new Spatial faulty signals are activated.

## 5. Case Study

### 5.1. Hanoi WDN

**Figure 1:**
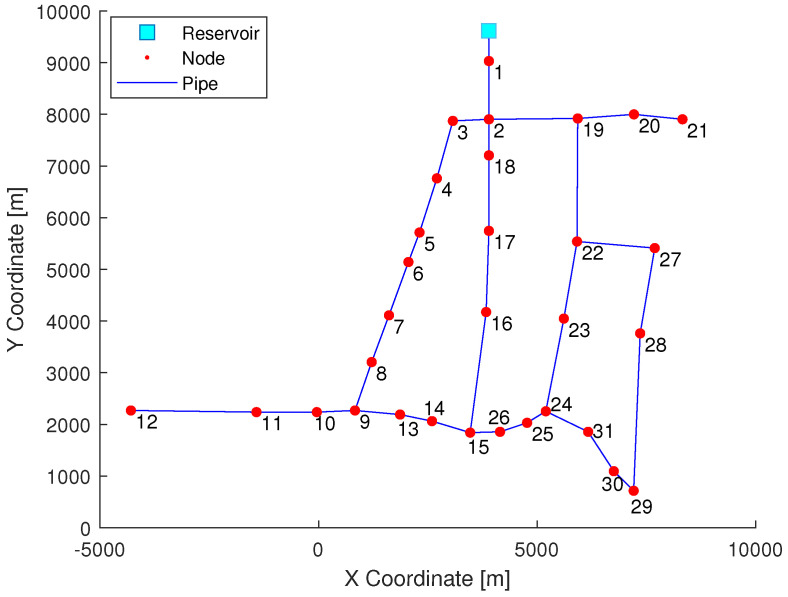
Simplified Hanoi topological WDN.

The Network used for the case study is a reduced city’s network model from Hanoi’s WDN (Vietnam). It is composed of one inlet (reservoir), 34 pipes, and 31 nodes, represented by Figure 1.

To analyze the performance of the proposed approach, data with different conditions have been generated artificially using the EPANET hydraulic simulator [29]. In order to consider realistic scenarios, some uncertainty has been added to the data [30]: the magnitude of the leak is random with a range of 25 to 75 [l/s], that is, between 1% and 2.5% of the average inlet flow of the WDN. Furthermore, white noise has been combined to emulate the noise present in real measurements, and uncertainty of 10% (uniform distribution) was added in the nominal demand value.

**Figure 2:**
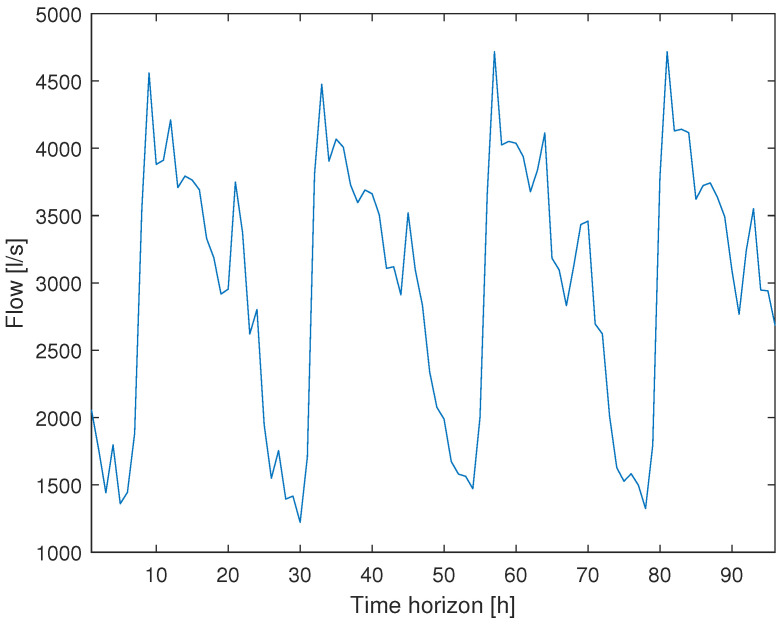
Flow consumption.

The daily water consumption pattern used for the calibration of Equation (6) is shown in Figure 2, having four days of operation.

The sample rate is 10 min, but average hourly measurements are calculated to reduce uncertainties on the diagnostic stage.

#### Results

The evaluation of the performance of the proposed leak localization method at node level defined in Equation (21) will be analyzed utilizing Average Topological Distance (ATD) [11]. The ATD represents the node’s distance between the node predicted as leaking and the actual node with the leak. To calculate the ATD, it is first necessary to create a matrix containing the minimum topological distance (in nodes or meters), $A\in R^{n-n_{I}\times n-n_{I}}$.

Finally, the confusion matrix $\Gamma_{i,j}\left(n-n_{I}\times n-n_{I}\right)$ defined in [18] and depicted in Table 1 is used to assess the performance of Equation (21). The rows of this matrix correspond to the leak scenario and the columns to where the leak is located ($\hat{l}$) by the leak localization method.

Considering the confusion matrix Γ, the ATD can be computed as follows:

(26) $$ATD=\frac{\sum_{i=1}^{n-n_{I}}\sum_{j=1}^{n-n_{I}}\Gamma_{i,j}A_{i,j}}{\sum_{i=1}^{n-n_{I}}\sum_{j=1}^{n-n_{I}}\Gamma_{i,j}}.$$

Four cases have been considered with different quantities of sensors in the network to analyze how this affects the final result. Table 2 presents the distribution of the selected nodes to contain a sensor. As seen in [31], the positioning of the sensors produces different results. As this work did not discuss the adequate sensors’ arrangement, they were chosen to consider an improvement in the results regarding the number of sensors.

Using the inlet flow data and non-leak historical pressure measurements of the selected sensors, the $\beta_{i}$ and the $\alpha_{i}$ with i=1,…,31 in (6) have been identified (notice that $n_{I}=1$). With these parameters, the pressure estimations under a non-leak condition in the network can be calculated considering inlet measurements using Equation (6) and posteriorly applied to calculate the residuals (9) with measured pressures in leak scenarios. In addition, non-leak pressure measurements and estimations are used to generate fault-free pressure residuals $r_{s_{i}}\left(k\right)$ and bounds $\underline{\sigma_{i}}$, ${\bar{\sigma }}_{i}$$i=1,\ldots ,n_{s}$, as well as spatial residuals $Sr_{s_{i},s_{j}}\left(k\right)$ and bounds ${\underline{\varepsilon }}_{i,j}$, ${\bar{\varepsilon }}_{i,j}$$\;\forall i=1,\ldots ,n_{s}-1\;\mathrm{and}\;j=i+1,\ldots ,n_{s}$.

**Figure 3:**
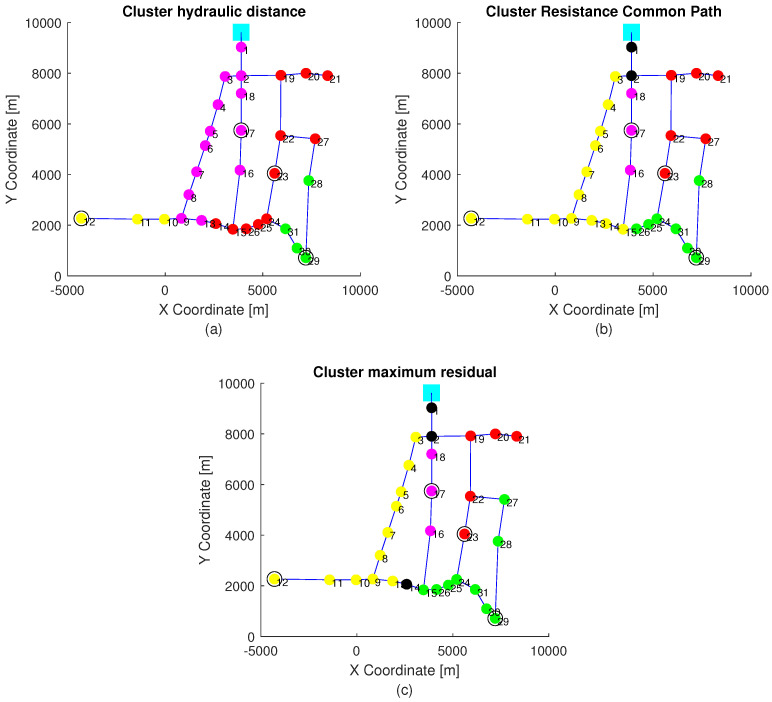
The $n_{s}$ clustering generated with the aspects: (**a**) shortest weighted pipe length, (**b**) The resistance takes into account the common path $R_{j,s_{i}}^{c}$; (**c**) is the maximum residual. Nodes in yellow, violet, red and green define the cluster related to sensor installed in node 12, 17, 23, and 29, respectively.

For every sensor configuration, normalized incidence factors (14) have been computed with topological information: node connections and pipe characteristics (length, diameter, and roughness). Figure 3 has the objective of comparing the information of the incidence of single leaks to pressure sensors obtained by a hydraulic model with the one obtained by means of topological information. The nodes selected to have sensors are the ones defined in the first case in Table 2. In particular, Figure 3c shows the clustering that groups the nodes that produce the highest effect in a specific pressure sensor. Nodes in yellow define the cluster of nodes where a leak produces a maximum pressure deviation from the non-leak scenario in the sensor installed in node 12, and the same is true for nodes in violet, red and green regarding pressure sensors in nodes 17, 23, and 29, respectively. Finally, nodes in black are nodes that produce a similar variation of pressure (difference of variation less than 0.1 [mwc]) in at least two different pressure sensors. In order to obtain this information, a hydraulic model to compute the difference of non-leak and leak pressures in all the nodes for the different leaks is required. On the other hand, Figure 3a shows the clustering that takes into account the shortest weighted pipe length (hydraulic distance), that is, the sum of $\left(L_{z}/D_{z}^{4.87}\right)$ for all edge $e_{z}$ in the path to the sensors, being the smallest one used to define the most resemblance to the sensor, used in Ref. [18]. Finally, the clustering depicted in Figure 3b is defined by Equation (17), which is based on the common resistance path explained in Section 3. These two last clusters that only require topological information could be used in the leak localization method at the cluster level defined in Equation (10). It is important to emphasize that the clustering based on the resistance common path, proposed in this paper and depicted in Figure 3b, resembles the clustering based on the actual leak effect in the network (given by the model) depicted in Figure 3c much more than the clustering based in the hydraulic distance depicted in Figure 3a. Therefore, the clustering proposed in this paper provides more accurate information for leak localization purposes than that based on the hydraulic distance. For example, as shown in Figure 3c, when a leak is present in nodes 3, 4, 5, 6, 7, 8 or 9, the sensor most affected by the leak is the sensor in node 12. This information is the same as the one provided by the clustering depicted in Figure 3b and is computed only with topological information. However, using the clustering of Figure 3a based on the hydraulic distance between nodes and sensors, the closest sensor to these nodes is the sensor in node 17.

**Figure 4:**
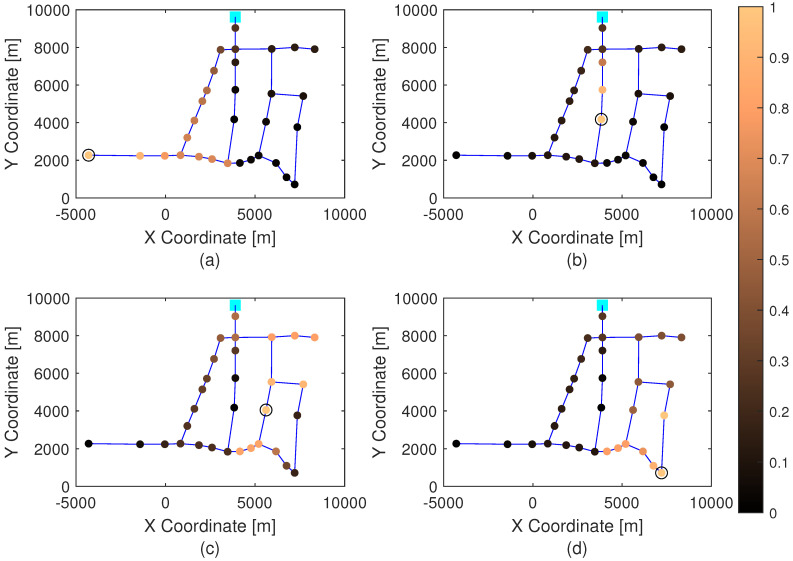
Relative incidence index $\lambda_{j,s_{i}}$ for all the nodes (j=1,…,31), corresponding to: (**a**) 1st sensor (i=1), (**b**) 2nd senor (i=2), (**c**) 3rd sensor (i=3), and (**d**) 4th sensor (i=4).

Figure 4 shows the correlation analyses of the relative incidence index $\lambda_{j,s_{i}}$ defined in Equation (14) for all the nodes j=1,…,31 depicted in every subplot for every sensor $s_{i}$i=1,…,4. As this index is normalized, its values are in the range [0,1). The nodes with the higher index (more brown color) are those that produce a higher effect in the pressure sensor $s_{i}$.

**Figure 5:**
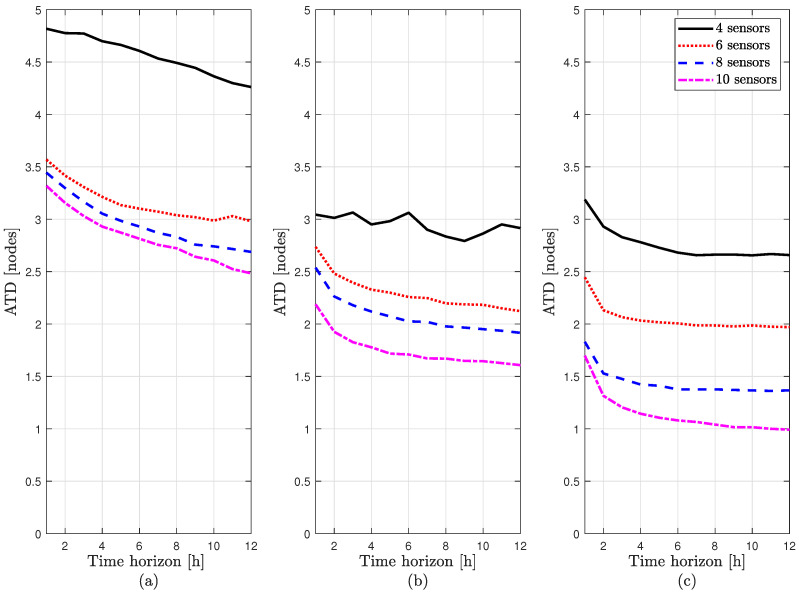
Evolution of the ATD between the methods: (**a**) using the Kriging interpolation method presented in [18], (**b**) using the new leak localization method with the same sensor configurations as in [18] and (**c**) using the new localization method with sensor configurations of Table 2.

Figure 5a displays the evolution of the ATD (in nodes) obtained by the leak localization method based on the Kriging spatial interpolation methodology presented in [18] with the time horizon (in hours) used recursively by the Bayes’ rule in (20). Four different sensor configurations are considered with 4, 6, 8, and 10 sensors placed optimally in order to maximize the performance of the leak localization proposed [18]. The performance can be compared with the one obtained by the new leak localization method proposed in this paper at node level defined in Equation (19) with the same dataset and the same sensor configurations as in [18], depicted in Figure 5b and with the sensor configurations shown in Table 2, depicted in Figure 5c.

Figure 5a shows that the leak detection performance of the Kriging method improves significantly from four to eight sensors and more moderately compared to ten sensors, still having a good result, even with noise data managing to reach an ATD equal to 2.5 node. When compared to the newly proposed leak localization method, as can be seen in Figure 5b,c, the new leak localization method always outperforms the Krigring method, even in the case of using the sensor configurations proposed in [18] that were computed to optimize the performance of the Kriging method. Figure 5c shows that the sensor configurations proposed in [18] are not optimal for the proposed method but the performance can be improved by changing the sensor configurations, in this case manually.

In order to illustrate the performance of the proposed sensor validation method, Case 1 (four sensors) will be considered. The four-sensor residuals computed by Equation (7) have been considered in a time window of 24 h using leak-free data leading to upper residual bounds equal to:

$$\left[{\bar{\sigma }}_{1},{\bar{\sigma }}_{2},{\bar{\sigma }}_{3},{\bar{\sigma }}_{4}\right]=\left[0.11,0.06,0.09,0.11\right]$$

and the lower residual bounds equal to:

$$\left[{\underline{\sigma }}_{1},{\underline{\sigma }}_{2},{\underline{\sigma }}_{3},{\underline{\sigma }}_{4}\right]=\left[-0.14,-0.10,-0.10,-0.08\right].$$

In the same way, the six spatial residuals defined by (23) have been computed in the same conditions as sensor residuals leading to spatial residual bounds:

$$\left[{\bar{\varepsilon }}_{1,2},{\bar{\varepsilon }}_{1,3},{\bar{\varepsilon }}_{1,4},{\bar{\varepsilon }}_{2,3},{\bar{\varepsilon }}_{2,4},{\bar{\varepsilon }}_{3,4}\right]=\left[0.06,0.07,0.08,0.04,0.05,0.03\right]$$

and

$$\left[{\underline{\varepsilon }}_{1,2},{\underline{\varepsilon }}_{1,3},{\underline{\varepsilon }}_{1,4},{\underline{\varepsilon }}_{2,3},{\underline{\varepsilon }}_{2,4},{\underline{\varepsilon }}_{3,4}\right]=\left[-0.04,-0.06,-0.08,-0.06,-0.09,-0.03\right].$$

**Figure 6:**
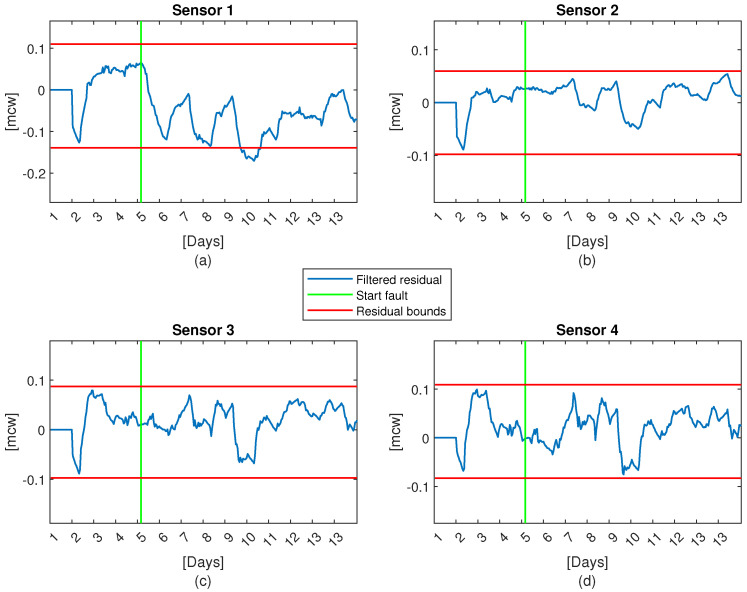
Graph of the filtered residual with a fault in sensor number 1 (**a**) 1st sensor, (**b**) 2nd senor, (**c**) 3rd sensor, and (**d**) 4th sensor.

**Figure 7:**
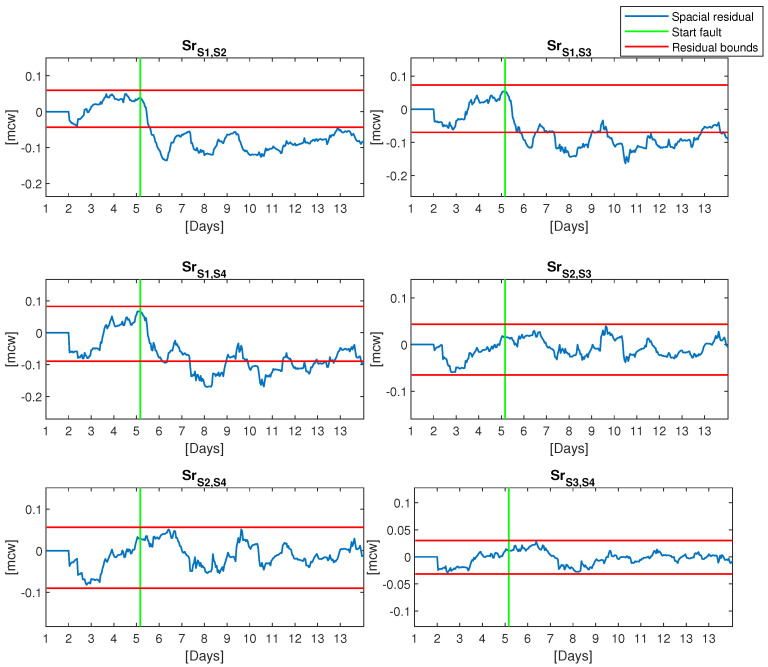
Graph of the spatial residual with a fault in sensor number 1.

Figure 6 and Figure 7 depict the evolution of sensor and spatial residuals with their respective residual bounds in a fault scenario of sensor 1 that corresponds to the pressure sensor in node 12. The fault is a drift of 0.1[mcw] that starts on the 5th day. As shown in Figure 6, by applying (22) to sensor residuals it is impossible to detect the fault until the end of the day 9 (i.e., 4 days later) when residual sensor 1 violates the bounds. However, by applying (24) to spatial residuals it is possible to detect the fault in 10 h: $Sr_{s_{1},s_{2}}$ violates its bounds in 10 h, and $Sr_{s_{1},s_{3}},Sr_{s_{1},s_{4}}$ violate their bounds in 16 h and 22 h, respectively.

### 5.2. Modena WDN

The second case study selected to test the performance is the reduced model of the real water distribution network of the Italian city Modena. This large-scale network is comprised of 268 junctions (nodes) connected through 317 pipes and served by four reservoirs. There are no pumps in the network since it is entirely gravity-fed [32,33].

The EPANET hydraulic simulator was used to generate artificial data to analyze the performance of the proposed method. The following simulation conditions were considered:

- The leak scenario consists of data samples collected every 10 min and filtered to hourly values to reduce the uncertainty in the data;
- The uncertainty of demand is considered by introducing the uncertainty of 10 [%] (normal distribution) of the nominal demand value. In addition, white noise is deemed to emulate the noise in the measurements;
- The leak size is randomly selected with a range of 3 to 6 [l/s], representing 1% to 2.5% of the network consumption.

The sensor bias, sensor drift, and abrupt sensor failure of sensor faults were proposed to analyze the sensor validation method. The sensor bias fault was simulated as a step change, and the drift fault was given as a time-varying ramp signal. In both cases, the fault magnitude was randomly chosen with a range of 0.1 to 0.2 [mwc]. The last fault was simulated by turning the sensor output to zero.

#### Results

**Figure 8:**
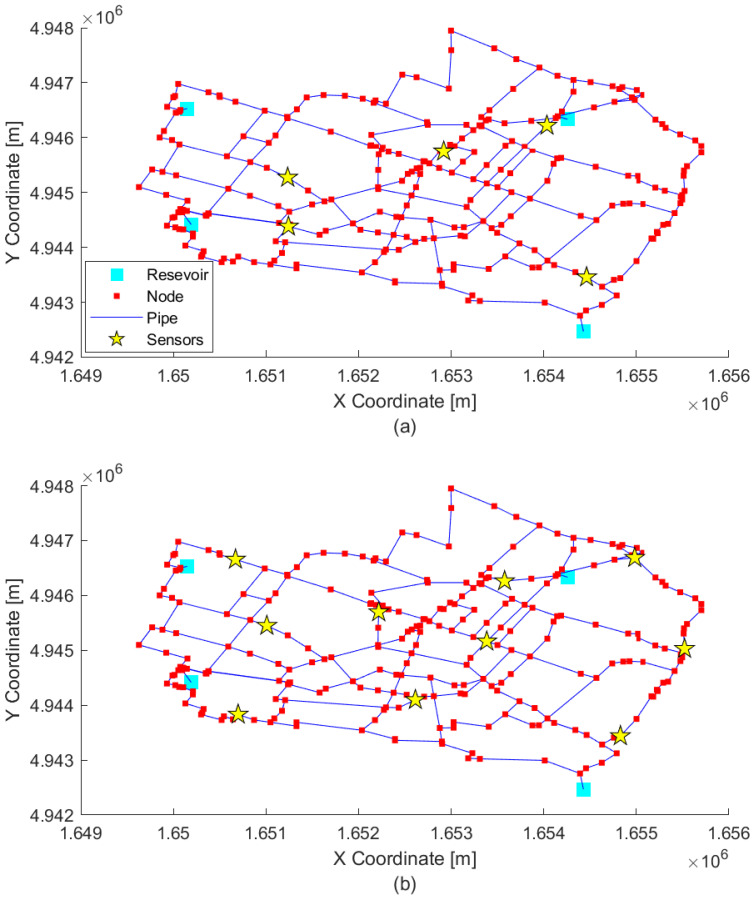
Configuration of pressure sensors in Modena WDN: (**a**) 5 sensors, (**b**) 10 sensors.

As applied in the previous case study, the Average Topological Distance (ATD) was used to assess the performance of the proposed leak localization method at the node level defined in (19). Two scenarios have been considered with five and ten pressure sensors that are presented in Figure 8a,b respectively. As emphasized in the last section, performance in the leak localization task is highly dependent on the number of sensors installed in the network [34,35,36].

**Figure 9:**
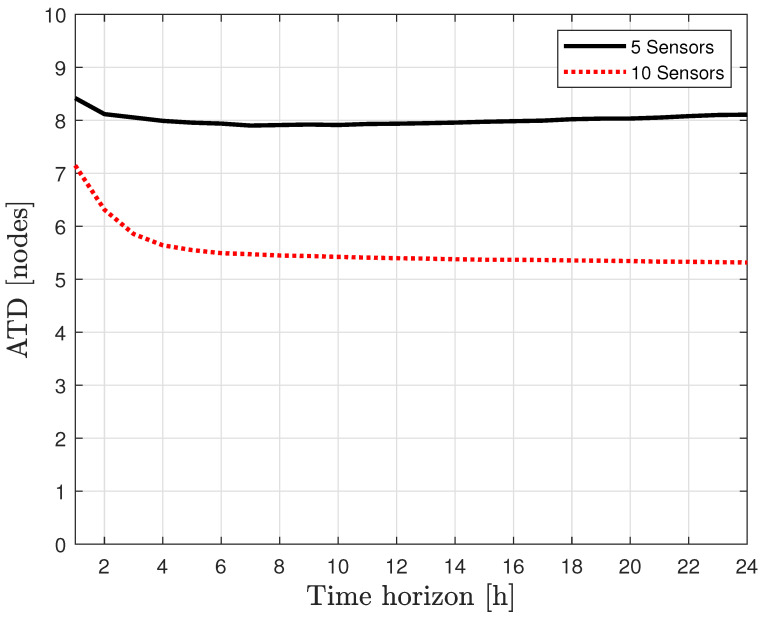
Evolution of the ATD.

Figure 9 shows the result of ATD evolution as defined in (26) applied with Bayes’ posterior time reasoning (20) to represent the leak location performance of the proposed method. This figure shows that the leak localization performance reached an ATD of 8 and 5.5 nodes with 5 and 10 inner pressure sensors installed in the network respectively. Considering that the proposed leak localization method only requires topological information and non-leak historical data in available measurements, the obtained performance is reasonably good.

On the other hand, a total of 6000 scenarios were simulated with 10 days each to evaluate the sensor validation method for the five sensor configurations depicted in Figure 8a. Thus, 1000 scenarios were generated for each sensor with sensor bias, sensor drift, and abrupt sensor failure applied randomly, and the remainder 1000 without faults.

To calculate the residual and spatial residuals bounds, a 6-month leak-free scenario was generated. The five sensor residuals computed by Equation (8) considering the time window of 24 h and increasing 24% observed bounds. Leading to upper residual bounds equal to:

$$\left[{\bar{\sigma }}_{1},{\bar{\sigma }}_{2},{\bar{\sigma }}_{3},{\bar{\sigma }}_{4},{\bar{\sigma }}_{5}\right]=\left[0.10,0.06,0.04,0.01,0.04\right]$$

and to lower residual bounds equal to:

$$\left[{\underline{\sigma }}_{1},{\underline{\sigma }}_{2},{\underline{\sigma }}_{3},{\underline{\sigma }}_{4},{\underline{\sigma }}_{5}\right]=\left[-0.08,-0.05,-0.03,-0.01,-0.06\right].$$

Following, the ten spatial residuals defined by (23) were computed in the same conditions as sensor residuals leading to spatial residual bounds:

$$\begin{array}{cc} {}[{\bar{\varepsilon }}_{1,2}, & {\bar{\varepsilon }}_{1,3},{\bar{\varepsilon }}_{1,4},{\bar{\varepsilon }}_{1,5},{\bar{\varepsilon }}_{2,3},{\bar{\varepsilon }}_{2,4},{\bar{\varepsilon }}_{2,5},{\bar{\varepsilon }}_{3,4},{\bar{\varepsilon }}_{3,5},{\bar{\varepsilon }}_{4,5}]=\\ \; & \;\left[0.07,0.08,0.08,0.10,0.06,0.05,0.06,0.03,0.06,0.05\right] \end{array}$$

and

$$\begin{array}{cc} {}[{\underline{\varepsilon }}_{1,2}, & {\underline{\varepsilon }}_{1,3},{\underline{\varepsilon }}_{1,4},{\underline{\varepsilon }}_{1,5},{\underline{\varepsilon }}_{2,3},{\underline{\varepsilon }}_{2,4},{\underline{\varepsilon }}_{2,5},{\underline{\varepsilon }}_{3,4},{\underline{\varepsilon }}_{3,5},{\underline{\varepsilon }}_{4,5}]=\\ \; & \;\left[-0.07,-0.09,-0.08,-0.09,-0.05,-0.04,-0.06,-0.03,-0.04,-0.04\right]. \end{array}$$

For this study, the evaluation metric applied was classification accuracy. To this purpose, the confusion matrix was used, which presents the classification accuracy and the misclassification error, and the horizontal axis of the confusion matrix describes the predicted labels of samples, while the longitudinal axis depicts the true labels of samples. The right side shows the percentages of correctly and incorrectly classified observations for each true class.

**Figure 10:**
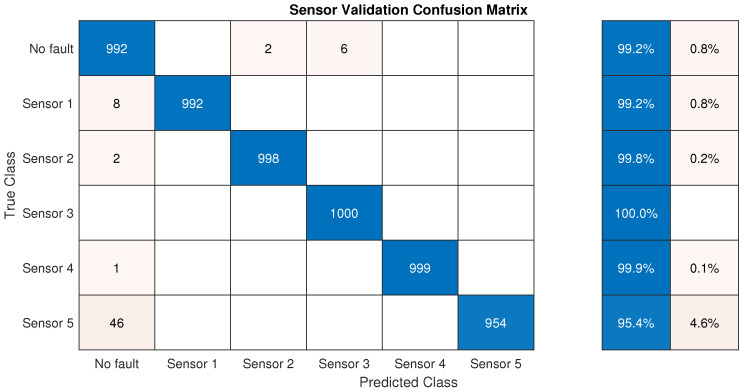
Confusion matrix for sensor validation method.

Figure 10 illustrates the result for the confusion matrix for all scenarios generated, and depicts that the accuracy of detecting faults in the sensor is very high, where the lowest accuracy is presented in fault sensor number five with an accuracy of 95.4% and the highest in fault sensor number three with 100% accuracy. Regarding the accuracy of the scenario with no-fault, eight of the 1000 fault free scenarios presented one false alarm among the 240 samples of the scenario; therefore providing an average interval between false detections of 240,000/8 = 30,000 h.

## 6. Conclusions

A new data-driven method for leak localization in WDN based on historical non-leak data and the topological information of the network is proposed. The proposed method is triggered when a leak is detected, and it is based on the evaluation of residuals generated by leak pressure measurements in some inner nodes and the estimation of leak-free pressures in these nodes utilizing a reduced-order model and historical data. Topological information is used to compute a new incidence factor that considers the most probable path of water from reservoirs to pressure sensors and potential leak nodes. The proposed incidence factor combined with residual information generates a likelihood index that allows leak localization at the node level. In addition, a sensor validation method based on the sensor pressure residuals, which is able to detect and isolate pressure sensor faults, is proposed.

The proposed method’s general performance for leak location and sensor validation is evaluated in reduced models of the Hanoi and Modena water distribution networks. The results of the leak localization are compared to another technique published with satisfactory results. Future works can be developed to improve the leak localization and sensor validation performances, with a study of an algorithm to automatically determine the optimal sensors required to maximize the leak localization performance.

## Figures and Tables

**Table 1:** Confusion matrix Γ.

	${\hat{l}}_{1}$	⋯	${\hat{l}}_{i}$	⋯	${\hat{l}}_{n-n_{I}}$
$l_{1}$	$\Gamma_{1,1}$	⋯	$\Gamma_{1,i}$	⋯	$\Gamma_{1,n-n_{I}}$
⋮	⋮	⋮	⋮	⋮	⋮
$l_{i}$	$\Gamma_{i,1}$	⋯	$\Gamma_{i,i}$	⋯	$\Gamma_{i,n-n_{I}}$
⋮	⋮	⋮	⋮	⋮	⋮
$l_{n-n_{I}}$	$\Gamma_{n-n_{I},1}$	⋯	$\Gamma_{n-n_{I},i}$	⋯	$\Gamma_{n-n_{I},n-n_{I}}$

**Table 2:** Nodes with sensors.

Case	Nodes with Sensors
1	12, 17, 23, 29
2	6, 12, 17, 23, 29, 21
3	6, 12, 15, 17, 23, 21, 27, 30
4	6, 9, 12, 15, 17, 24, 21, 22, 28, 29, 31

## Data Availability

The data can be found in https://github.com/adeboracris/Epanet_Leak_localization (accessed on 10 November 2021).
